# Large-area, continuous and high electrical performances of bilayer to few layers MoS_2_ fabricated by RF sputtering via post-deposition annealing method

**DOI:** 10.1038/srep30791

**Published:** 2016-08-05

**Authors:** Sajjad Hussain, Jai Singh, Dhanasekaran Vikraman, Arun Kumar Singh, Muhammad Zahir Iqbal, Muhammad Farooq Khan, Pushpendra Kumar, Dong-Chul Choi, Wooseok Song, Ki-Seok An, Jonghwa Eom, Wan-Gyu Lee, Jongwan Jung

**Affiliations:** 1 Graphene Research Institute, Sejong University, Seoul 143-747, Korea; 2 Faculty of Nanotechnology & Advanced Materials Engineering and Graphene Research Institute, Sejong University, Seoul 143-747, Korea; 3 Dr. H. S. Gour Central University, Sagar, M.P. 470003, India; 4 Division of Energy Systems Research, Ajou University, Suwon 443-749, Republic of Korea; 5 Department of Physics and Graphene Research Institute, Sejong University, Seoul 143-747, Korea; 6 Institute of Atomic and Molecular Sciences, Academia Sinica, Taipei, 10617, Taiwan; 7 Thin Film Materials Research Group, Korea Research Institute of Chemical Technology, Daejon 305-600, Korea; 8 National Nano Fab Center, Daejeon, Korea

## Abstract

We report a simple and mass-scalable approach for thin MoS_2_ films via RF sputtering combined with the post-deposition annealing process. We have prepared as-sputtered film using a MoS_2_ target in the sputtering system. The as-sputtered film was subjected to post-deposition annealing to improve crystalline quality at 700 °C in a sulfur and argon environment. The analysis confirmed the growth of continuous bilayer to few-layer MoS_2_ film. The mobility value of ~29 cm^2^/Vs and current on/off ratio on the order of ~10^4^ were obtained for bilayer MoS_2_. The mobility increased up to ~173–181 cm^2^/Vs, respectively, for few-layer MoS_2_. The mobility of our bilayer MoS_2_ FETs is larger than any previously reported values of single to bilayer MoS_2_ grown on SiO_2_/Si substrate with a SiO_2_ gate oxide. Moreover, our few-layer MoS_2_ FETs exhibited the highest mobility value ever reported for any MoS_2_ FETs with a SiO_2_ gate oxide. It is presumed that the high mobility behavior of our film could be attributed to low charged impurities of our film and dielectric screening effect by an interfacial MoO_x_Si_y_ layer. The combined preparation route of RF sputtering and post-deposition annealing process opens up the novel possibility of mass and batch production of MoS_2_ film.

Recently, MoS_2_ has attracted tremendous interest due to its film thickness scalability, its reducibility from bulk to a monolayer without surface dangling bonds or native oxides, and its promising carrier transport properties^1^,^2^. In contrast to graphene, which is intrinsically a semimetal with a zero band-gap, MoS_2_ is a semiconductor, which makes it a suitable substrate material for 2-dimensional (2D) field effect transistors (FETs)^3^,^4^. From an application point of view, a mass-producible growth technique for large-area, continuous, and high-quality MoS_2_ film on dielectrics is a pre-requisite. Micromechanical exfoliation method provides the purest MoS_2_ flakes with the highest material quality, the sample size is extremely limited^2^,^5^. Several attempts have performed by different groups to satisfy those needs for MoS_2_ film^6^. Many research groups also have reported promising growth route of CVD- MoS_2_^7^,^8^,^9^. Sulfurization of molybdenum (Mo)^10^,^11^ and thermolysis of Mo compounds^10^,^12^ and (NH_4_)_2_MoS_4_^13^ have attempted previously for preparation MoS_2_. MoO_3_ and MoCl_5_ along with sulfur are common precursors for MoS_2_-CVD^14^,^15^,^16^,^17^. Such methods usually yielded multilayer and suffered due to non-uniform film thickness and low carrier mobility^10^,^14^,^15^,^18^,^19^. Moreover, the synthesized continuous MoS_2_ films via the surface treatment exhibits very low carrier mobility (0.02–7 cm^2^/Vs)^10^,^14^,^15^,^18^,^20^. Continuous CVD-MoS_2_ films have been demonstrated using MoCl_5_ without pre-treatment, but the reported carrier mobility is also very low (0.003–0.03 cm^2^/Vs)^18^. Sanne *et al*.^21^ reported mobility value of 24 cm^2^/Vs and I_on_/I_off_ current ratio exceeding 10^7^ for top-gated MoS_2_ FETs with high-k gate dielectric on Si_3_N_4_. Ma *et al*.^22^ demonstrated the vapor-solid growth of few-layer MoS_2_ films on (0001) oriented sapphire. They estimated room temperature mobility of 192 cm^2^/Vs from the space-charge limited transport regime of the film. Laskar *et al*.^23^ attained large-area MoS_2_ films on (0001) oriented sapphire using sulfurization of e-beam evaporated Mo. They reported field-effect mobility of ~12 cm^2^/Vs using Mott-Guirney law with the carrier density of 10^16^ cm^−3^. Still, the lack of pristine quality, and wafer-scale synthesis of continuous MoS_2_ film on SiO_2_ is a challenging issue to be addressed.

Recently, there are few attempts to revive the sputtering technique for the growth of thin MoS_2_ film^24^,^25^,^26^. However, the reported films are either relatively thick or the reported electrical and optical properties are rare and poor^27^,^28^,^29^. Muratore *et al*.^27^ and Qin *et al*.^28^ reported the synthesis of continuous few-layer MoS_2_ by sputtering method using a MoS_2_ target. Tao *et al*.^30^ reported MoS_2_ film using Mo target sputtered in vaporized sulfur ambient, but the grown MoS_2_ film also exhibited p-type behavior with hole mobility up to ~12.2 cm^2^/Vs and low on/off current ratio of ~10^3^.

Herein, we report a simple and mass-scalable approach for thin MoS_2_ films via MoS_2_-RF sputtering combined with the post-deposition annealing process for the first time. From Raman spectra and photoluminescence (PL), it has been shown that the crystalline quality of the as-sputtered MoS_2_ films was highly enhanced through the post-deposition annealing process. Synthesized bilayer MoS_2_ films exhibited high field-effect mobility of ~29 cm^2^/Vs and a current on/off ratio of ~10^4^. The mobility increased up to ~173–181 cm^2^/Vs, respectively, for few-layer MoS_2_ films. To the best of our knowledge, the mobility value of our bilayer MoS_2_ FETs is larger than any reported results of single to bilayer MoS_2_ FETs grown on SiO_2_/Si with a SiO_2_ gate oxide. Furthermore, the mobility value (~173–181 cm^2^/Vs) of our few-layer MoS_2_ FETs is the highest ever for any MoS_2_ FETs with a SiO_2_ gate oxide. It is much higher than that of single crystal exfoliated MoS_2_ flakes on SiO_2_/Si substrate^31^ and comparable to the value of bulk MoS_2_, room temperature mobility limited by phonon-scattering^32^.

## Results and Discussion

**Figure 1:**
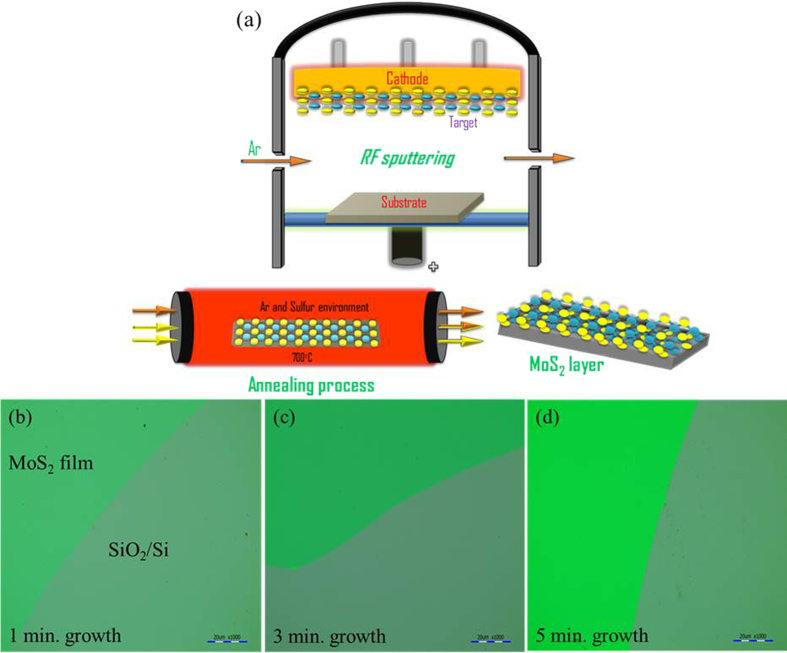
(**a**) Schematic representation of the experimental set-up. The RF sputtering technique was used to prepare as-sputtered MoS_2_ layer. Post-deposition annealing treatment was performed to further enhance crystalline quality in as-sputtered MoS_2_ under Ar and sulfur environment. Optical images of MoS_2_ films grown on SiO_2_/Si substrate. **(b)** MoS_2_ sputtered for 1 min; **(c)** MoS_2_ sputtered for 3 min; and **(d)** MoS_2_ sputtered for 5 min.

MoS_2_ films of different thicknesses were deposited by adjusting RF magnetron sputtering time such as 1, 3, 5 and 15 min onto SiO_2_/Si, quartz and sapphire substrates. The substrate temperature was varied from RT to 500 °C. As-sputtered films were subjected to post-deposition annealing treatment at 700 °C in the sulfur and Ar environment to improve their crystallinity. The detailed scheme for preparation and annealing processes is illustrated in Fig. 1(a). Optical microcopy images of sulfurized MoS_2_ films at 1, 3 and 5 min. sputtered on SiO_2_/Si substrate are shown in Fig. 1b–d.

**Figure 2:**
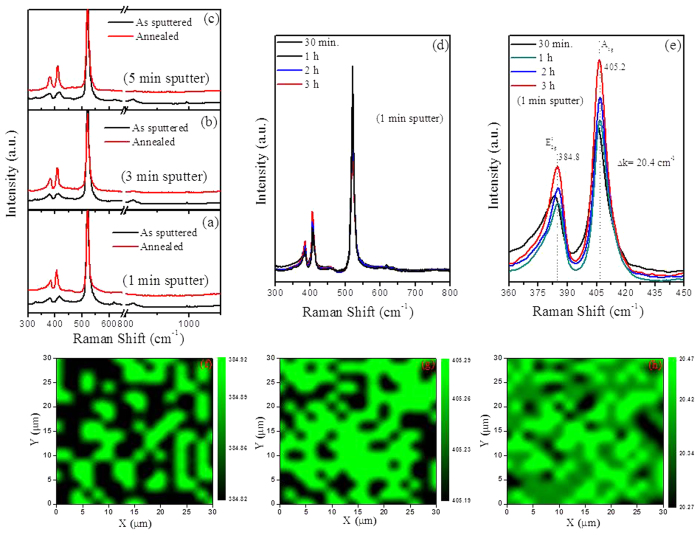
**(a–c)** Raman spectra of as-sputtered and annealed MoS_2_ films; **(d)** Raman spectra of MoS_2_ films annealed at different times of 30 min, 1 hour, 2 hours and 3 hours; **(e)** Magnified view of Raman spectra of figure (**d**); **(f–h)** Raman mapping for 1-min sample (30 μm × 30 μm). **(f)** E_2g_^1^ mode appears at 384.82–384.92 cm^−1^ (with a standard deviation 0.048 cm^−1^) **(g)** A_1g_ mode appears at 405.19–405.29 cm^−1^ (with a standard deviation 0.049 cm^−1^) **(h)** The measured frequencies difference (∆k) is in the range of 20.27–20.47 cm^−1^ (with a standard deviation 0.066 cm^−1^).

Raman spectra of the as-sputtered MoS_2_ films are shown in Fig. 2a–c. The as-sputtered MoS_2_ films exhibit the E^1^_2g_ and A_1g_ mode peaks with low intensity. It might be due to low crystalline quality and the presence of defects contributes to the broad and low intensity of the peaks. The strong substrate related peak is observed at 520 cm^−1^. As the sputter time increases, the Raman scattering peak intensities are slightly enhanced. Additional peaks at ~820 and ~992 cm^−1^ are related to the oxygen bonds and characteristic peaks of MoO_3_ (alpha(α)-MoO_3_)^33^. The symmetric stretch of 820 cm^−1^ (A_g_, B^1^_g_) is a terminal Mo = O bond and the 995 cm^−1^ (A_g_, B^1^_g_) is an asymmetric stretch of the terminal Mo = O bond along the a- and b-axes^24^,^25^,^34^. MoS_2_ films are highly sensitive to moisture and oxidize easily. It has been also proposed that conventional sputter-deposited MoS_2_ film contains oxygen substituted for sulfur atoms in the MoS_2_ crystal lattice during film growth^26^.

Figure 2a–c shows that Raman spectra variation through post-deposition annealing. The Raman peak enhancement indicates that the high-temperature annealing in the presence of sulfur and Ar greatly improved the crystallinity of as-sputtered MoS_2_ film. Moreover, MoO_3_-related peaks were significantly suppressed for the annealed MoS_2_ films. Through the post-deposition annealing in sulfur and Ar, the MoO_3_ is believed to be transformed into a crystalline MoS_2_ structure^10^,^35^. For the 1 min-sample (MoS_2_ sputtered for 1 min and annealed at 700 °C for 1 hour), the Raman peak difference between E^1^_2g_ and A_1g_ mode is ~20.5 cm^−1^, which is close to that of the exfoliated bilayer MoS_2_^36^. Figure 2d,e shows the Raman spectra according to the different annealing times from 30 min to 3 hours. The peak intensities are increased slightly with increase of annealing time. In order to focus oxygen-related peaks more precisely, the Raman analysis was performed for thick MoS_2_ films; as-sputtered films for 15 min at RT and 400 °C, and annealed MoS_2_ films (Figure S1). The thick film sputtered at RT exhibited strong MoO_3_ peaks at ~822 and ~992 cm^−1^ (Figure S1c,d). The oxygen peak intensities were reduced at higher substrate temperature (400 °C), but decreased most through the post-deposition annealing at 700 °C (the as-synthesized film was originally sputtered at RT). Raman mapping was performed over an area of 30 μm × 30 μm for 1 min-sample as shown in Fig. 2f–h. The E^1^_2g_ and A_1g_ mode peaks appear at ~384.82–384.92 (with a standard deviation 0.048 cm^−1^) and ~405.19–405.29 cm^−1^ (with a standard deviation 0.049 cm^−1^), respectively. The peak difference (∆k) values are in the range of ~20.27–20.47 cm^−1^ (with a standard deviation 0.066 cm^−1^), corresponding to the MoS_2_ bilayer^14^,^36^. For the 3 min-sample (MoS_2_ sputtered for 3 min and annealed at 700 °C for 1 hour, Figure S2), E^1^_2g_ and A_1g_ mode are located in the range of ~382.23–382.33 cm^−1^ (with a standard deviation 0.05 cm^−1^) and ~407.29–407.39 cm^−1^ (with a standard deviation 0.045 cm^−1^), respectively, with ∆k values in the range of ~24.96–25.16 cm^−1^ (with a standard deviation 0.066 cm^−1^), corresponding to few-layer MoS_2_ film^36^. For the 5 min-sample (MoS_2_ sputtered for 5 min and annealed at 700 °C for 1 hour), the E^1^_2g_ mode position downshifted to ~380.63–380.73 cm^−1^ (with a standard deviation 0.05 cm^−1^) and the A_1g_ mode upshifted to ~408.29–408.39 cm^−1^ (with a standard deviation 0.047cm^−1^). The ∆k value is increased to ~27.56–27.76 cm^−1^ (with a standard deviation 0.070 cm^−1^), suggesting that film thickness increment. The Raman measurement was also performed for as-synthesized MoS_2_ sputtered at various substrate temperatures from 200 to 500 °C (Figure S3). The as-sputtered film at a substrate temperature of 200 °C exhibits two characteristic MoS_2_ Raman peaks with low intensity (E^1^_2g_ mode at ~381 cm^−1^ and A_1g_ mode at ~411 cm^−1^). At higher substrate temperatures of 300, 400 and 500 °C, the Raman peak intensities are slightly varied and MoO_3_ peaks at ~822 and ~993 cm^−1^ are reduced. XRD was performed to investigate the structural properties of MoS_2_ film. XRD patterns of as-sputtered MoS_2_ thin films and the corresponding annealed films are shown in Figure S4a–c. For as-sputtered films, only a silicon substrate-related peak at 2θ = 33° is observed, supporting the amorphous structure of RT-sputtered MoS_2_ film. However, (002) lattice oriented diffraction line is observed at 2θ = 14.2° for annealed MoS_2_ films. The strong (002) peak is present when the periodicity in c-axis is normal to the MoS_2_ film plane which is in good agreement with the previous results^37^,^38^. As-sputtered MoS_2_ films sputtered at higher substrate temperatures revealed a very weak (002) peak and intensity tends to increase with the increase of sputtering temperature from 200 °C to 500 °C(Figure S5). Thus, Raman and XRD analysis revealed that increase of sputtering temperature improves the film quality and reduces oxygen content but is not sufficient for obtaining high quality MoS_2_ film; post-deposition annealing improves film quality the most.

**Figure 3:**
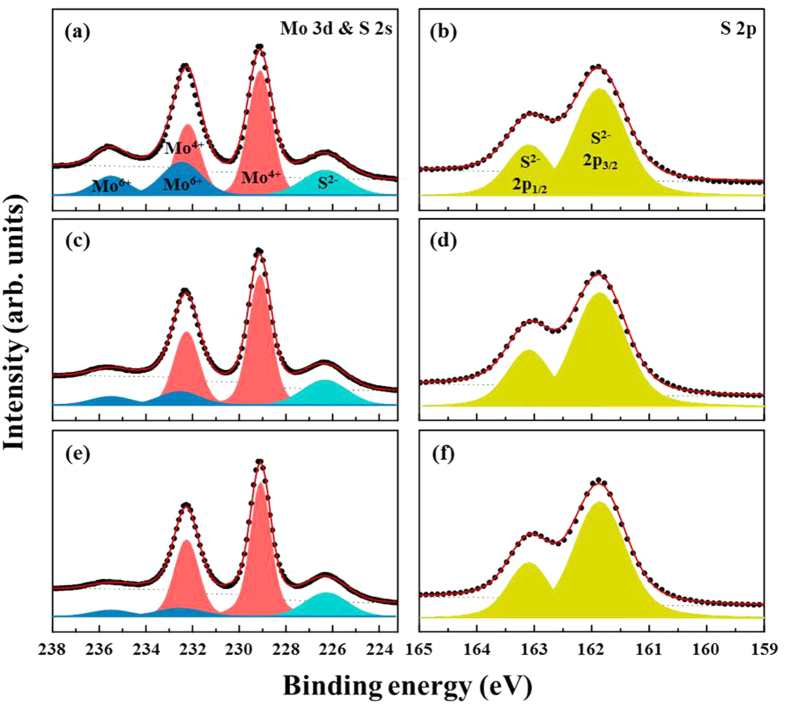
XPS spectra of MoS_2_ films annealed at 700 °C. Mo and S atoms binding energy spectra for different sputter time: (**a,b**) 1 min, (**c,d**) 3 min, and (**e,f**) 5 min.

XPS analysis was used to measure binding energies of Mo and S atom. For the 1 min-sample, Mo 3d peaks at 229.1 and 232.2 eV are exhibited (Fig. 3a), which is attributed to the doublet of Mo 3d_5/2_ and Mo 3d_3/2_, respectively^39^. Also sulfur atoms-related 2S pathetic peak is observed at 226.3 eV. S^2−^ peaks are also observed (Fig. 3b) at 161.9 and 163.1 eV due to S 2p_1/2_ and S 2p_3/2_, respectively. In addition, a peak at 235.9 eV corresponds to the Mo^6+^ of MoO_3_^40^. For the 3 min and 5 min-sample, the observed peaks are slightly shifted to lower binding energies, which may be due to the increment of the number of layers. All these results are in good agreement with the reported values for MoS_2_ crystal^41^. The intensity of Mo^6+^ peaks decreased with increasing growth time. The Mo^6+^ peaks indicate that some oxygen is incorporated in the grown MoS_2_ film. Oxygen can be incorporated as substitutional atoms at sulfur sites^42^, as atoms bound to Mo atoms at plane edges^26^, as an intercalant between basal planes as O_2_ or moisture (H_2_O)^43^, or as an interfacial Mo-oxide layer due to Mo-oxygen bonding at the MoS_2_-SiO_2_ interface^27^,^28^. XPS survey spectra of Figure S7 show that the total oxygen and silicon signal decreases with increasing sputtering time. This could be explained as the probability of electrons escaping from the SiO_2_ substrate reduces exponentially with increasing MoS_2_ thickness^31^.

**Figure 4:**
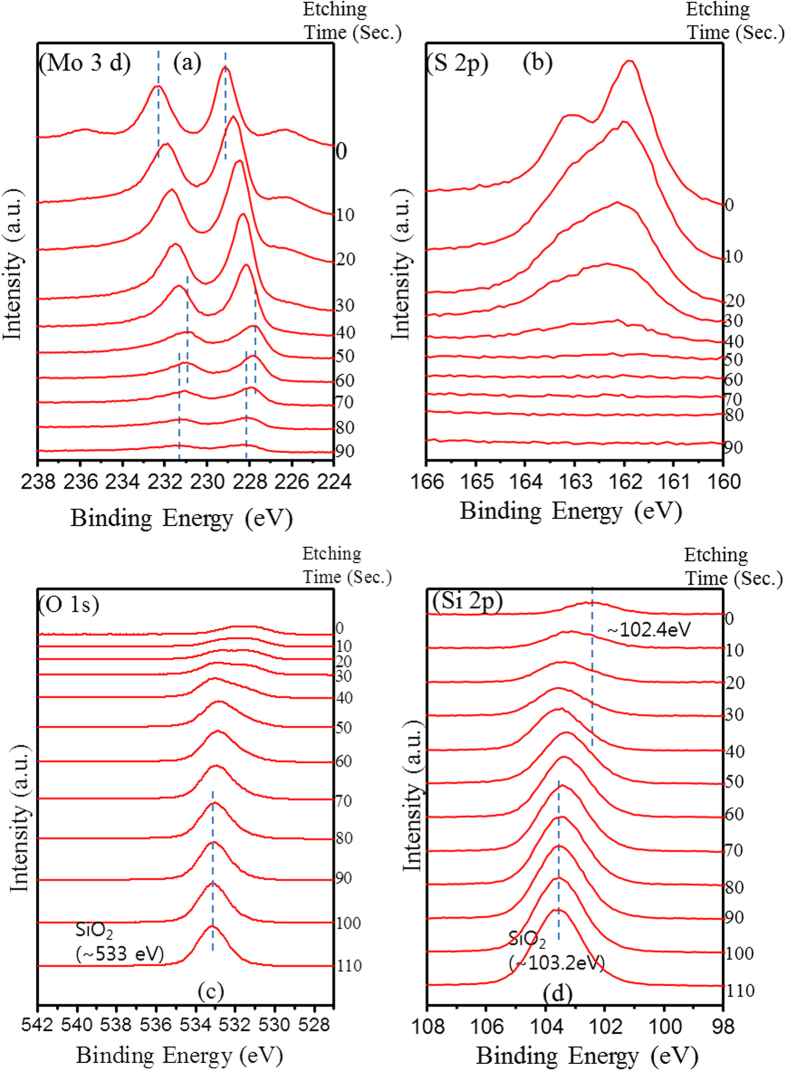
XPS Depth profile of few-layer MoS_2_ (5 min-sample). **(a)** Mo 3d core peaks as a function of etching time. Binding energies at 229.1 and 232.2 eV are associated with Mo^4+^ 3d5/2 and 3d3/2 core levels in MoS_2_, respectively, while S 2s appears at 226.3 eV. The peak at 235.9 eV indicates the presence of Mo^6+^ (MoO_3_) on the surface of the film. **(b)** Sulfur related S^2−^ peak change with etching time. The sulfur related peaks are eventually disappeared after 50~60 sec. **(c**,**d)** O 1s and S 2p peak depth profile with the etching time.

XPS depth profile analysis was performed to investigate the interfacial structure of the MoS_2_/SiO_2_ film. A 1keV Ar ion beam was used for sputtering purpose. XPS survey spectra depict that increment of oxygen peak as well as decrement of Mo core level peak with the increase of etching time (Figure S8c). The expanded view of Mo 3d core peak variations are displayed in the Fig. 4a as a function of etching time. Before the sputter etching, the peaks of Mo^4+^ 3d states are the main part of the spectra, and a small amount of MoO_3_ state exists on the surface. When the film is etched by ion beam, there is a chemical shift of its binding energy toward smaller values. The shift is attributed to the change in the chemical states of Mo^4+^ from the film surface to inner^44^. The Mo^6+^ peak of MoO_3_ is highly suppressed after etching for 10 sec. So, the Mo^6+^ peaks are mainly originated from the surface oxidation of MoS_2_. The peak shift proceeds until 60 sec. After 60 sec, the binding energy shifts back toward higher values. From the Fig. 4b, sulphur related S^2−^ peaks are decreased and broadened as etching proceeds due to the damage induced by Ar etching, and the peaks almost disappear after etching for 50~60 sec (Fig. 4b, Figure S8c, Supporting Information). On the contrary, Mo peaks still exist after 60 sec. Hence, it is highly likely that these Mo could be combined with oxygen atoms or Si atoms in SiO_2_ and form as a molybdenum oxide (MoO_x_), or molybdenum silicon oxide (MoO_x_Si_y_) layer. The Si 2p peak in Fig. 4d is exhibited at ~102 eV before Ar etching, and it upshifts towards ~103.2 eV, which is the binding energy of SiO_2_. It is suspected that the Si 2p peak at ~102 eV is due to the MoO_x_-SiO_x_ bonding^45^. The Si 2p binding energy at ~102 eV is very close to that of (MoO_3_)70(SiO)30 (102.5eV)^45^.

We later discuss that the interfacial layer can alter the electrical properties of MoS_2_ film. The XPS depth profiling was also performed for a very thick MoS_2_ film (Figure S8) and observed results are also similar to few-layer MoS_2_.

**Figure 5:**
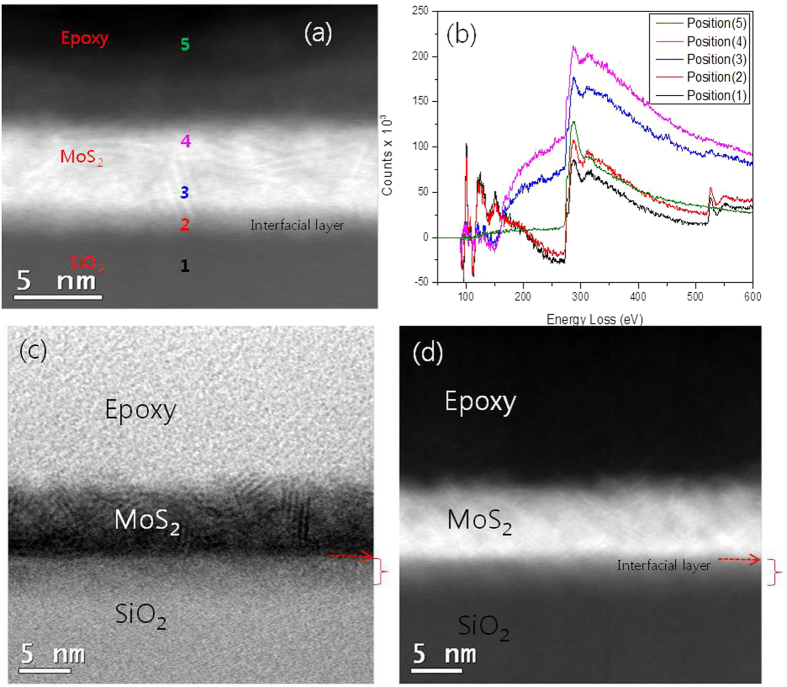
(**a**) HAADF image for few-layer MoS_2_ film (5min-sample) (**b**) EELS spectra evaluated at different depth positions of film which is labelled as 1,2,3,4 and 5 (**c**) HRTEM and (**d**) STEM-HAADF image.

Figure 5a,b shows the cross-sectional high-angle annular dark-field (HAADF) image and the corresponding electron energy loss spectroscopy (EELS) spectra for 5 min-sample. For the position 1 and position 2, ‘Si’ and ‘SiO_2_’ are detected at ~99 eV and ~105 eV, respectively, and ‘O’ is detected at ~525 eV. Therefore these two points are clearly SiO_2_. A sulfur is detected at ~160 eV from the region 3 and 4, and not from the position 1, 2 and 5, indicating that point 3 and 4 are MoS_2_. It is thought that position 2 looks bright due to higher scattering of Mo. The position 5 is an epoxy material exhibiting only C spectrum. The comparison of bright field and HAADF image (Fig. 5c,d) indicates that the region 2 is an interfacial layer of the MoS_2_/SiO_2_. It is suspected that during sputtering process, Mo adlayers are initially formed at the interface of MoS_2_/SiO_2_ and the Mo layers diffused into SiO_2_ during the annealing step, resulting in the formation of MoO_x_Si_y_ layer. The diffused interfacial layer appears brighter due to higher scattering with heavier atoms in that region than that in pure SiO_2_ film.

Luminescence properties were studied by PL analysis as shown in Figure S10. The PL peaks are very weak and broad for the as-sputtered films. As sputtering time increases, peak position is shifted to a higher wavelength since the film thickness increases^46^,^47^. The luminescence peak intensities are significantly increased for the annealed MoS_2_ films (Figure S10b). For the 1 min-sample, the major peak is located at ~662 nm (1.87 eV, A peak) and one minor peak at ~620 nm (2 eV, B peak), which corresponds to a direct excitonic transition at the K point of the Brillouin zone of MoS_2_. The energy difference (~0.13 eV) is due to the degeneracy breaking of the valence band, which is in a close agreement with the literature^48^,^49^. The measured FWHM value for direct transition of peak A is ~67 meV, which is similar to freely suspended samples of MoS_2_ (50–60 meV)^50^ and narrower than that of MoS_2_ exfoliated onto SiO_2_ (100~150 meV)^51^. The emission intensity gradually increases with red shift^52^,^53^ as increase of annealing time as shown in Figure S10c. This strong luminescence behavior is due to bilayer MoS_2_ with a highly crystalline structure and support our earlier observation by Raman and XRD analysis that crystalline quality improvement via annealing at 700 °C.

**Figure 6:**
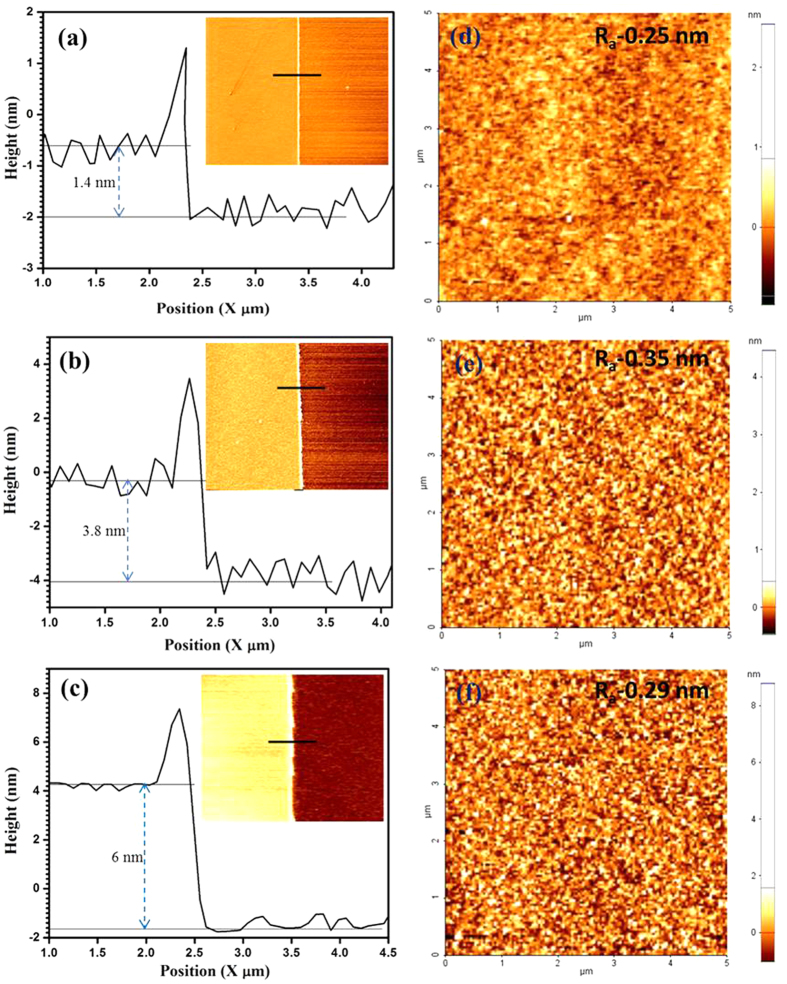
(**a–c**) AFM height profiles of annealed MoS_2_ films sputtered at 1, 3, and 5 min. Inset figure: 2D cross sectional images of the corresponding annealed MoS_2_ films; (**d**–**f**) Topographical images of annealed MoS_2_ films sputtered at 1, 3, and 5 min.

The thickness of the film was analyzed by AFM as shown in Fig. 6a–c. AFM scan was taken at a corner of the MoS_2_ film patterned using photolithography and etching process. For the 1 min-sample, the estimated thickness is ~1.4 nm, which is approximately close to bilayer MoS_2_^18^,^36^ (Fig. 6a). The thickness is ~3.8 nm (~5–6 layers) and ~6 nm (~8–10 layers), for the 3 min and 5 min-samples, respectively. Film continuity and uniformity were explored by AFM topographical 2D images. The surface roughness (R_a_, average deviation) values over a scanned area of 5 μm × 5 μm are ~0.18 nm, 0.22 nm, ~0.19 nm for 1, 3, and 5 min as-sputtered MoS_2_ films, respectively (Figure S11). 2D topographical images of the annealed films are shown in Fig. 6(d–f). The surface roughness (Ra) values are ~0.25 nm, ~0.35 nm, and ~0.29 nm for 1, 3, and 5 min-sample, respectively. These low roughness values support the highly uniform and continuous MoS_2_ films. We believe that a wafer-scale MoS_2_ could be produced by optimizing the sputtering time and annealing process.

**Figure 7:**
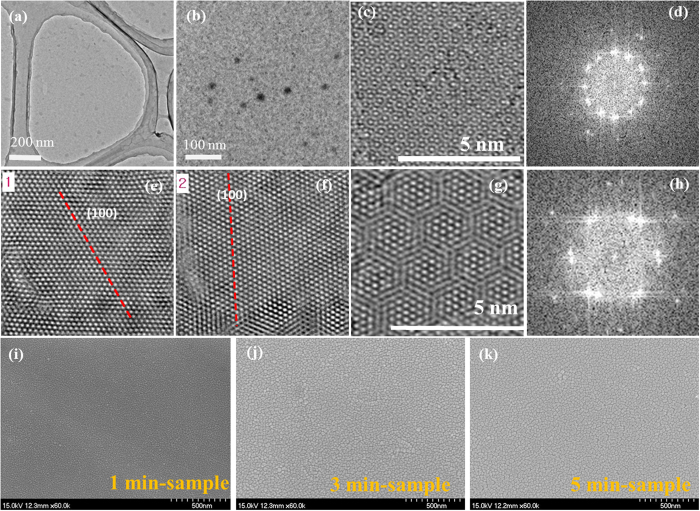
HRTEM images of 1 min-sample. **(a,b)** Low-magnification TEM image; **(c)** Moiré pattern of a bilayer-MoS_2_ area; **(d)** Fast Fourier transformation (FFT) image corresponding to the TEM image (**c**) supporting a bilayer MoS_2_ film; **(e,f)** Inverse FFT images of (**d**) showing the two layers are not Bernal-stacked, but rotated by ~26^o^; **(g)** Moiré pattern of a region in which two layers are stacked in a low rotation angle; **(h)** FFT image corresponding to the TEM image (**g**); (**i–k**) FE-SEM images of annealed MoS_2_ films sputtered at 1, 3, and 5 min.

HRTEM analysis was performed to explore the crystalline structure of MoS_2_ film (1 min-sample) as shown in Fig. 7. The lower magnification-HRTEM images are exhibited in Fig. 7a,b for a continuous MoS_2_ film on the copper grid. Figure S12a shows the HRTEM image over an area of 39 nm × 30 nm for 1 min-sample. The film shows a continuous film with a hexagonal lattice structure. Several types of Moiré fringes are observed and the film consists of mainly bilayer. The observed Moiré fringes in unfolded areas indicate that layers are not Bernal-stacked. A typical Moiré fringes (type B) in Figure S12 were analyzed using fast Fourier transformation (FFT) in Fig. 7d. The exhibited two inverse FFT images (Fig. 7e,f) are extracted from Figure 7c, showing that the two layers are rotated by ~26°. Figure 7g shows a different Moiré pattern (type A in Figure S12) consisting of two layers stacked in a low rotation angle, and the corresponding FFT image is shown in Fig. 7h. The continuous and uniform surface homogeneity was confirmed by FESEM images for 1, 3 and 5-min MoS_2_ samples as shown in Fig. 7(i–k), respectively. A monolayer is also spotted in Figure S12 (type C). Figure S12b,c shows HRTEM images for the 3 min and 5 min-sample as a supporting information. Large area MoS_2_ films with ~1 × 9 cm^2^ area and its Raman spectra are shown in Figure S13.

**Figure 8:**
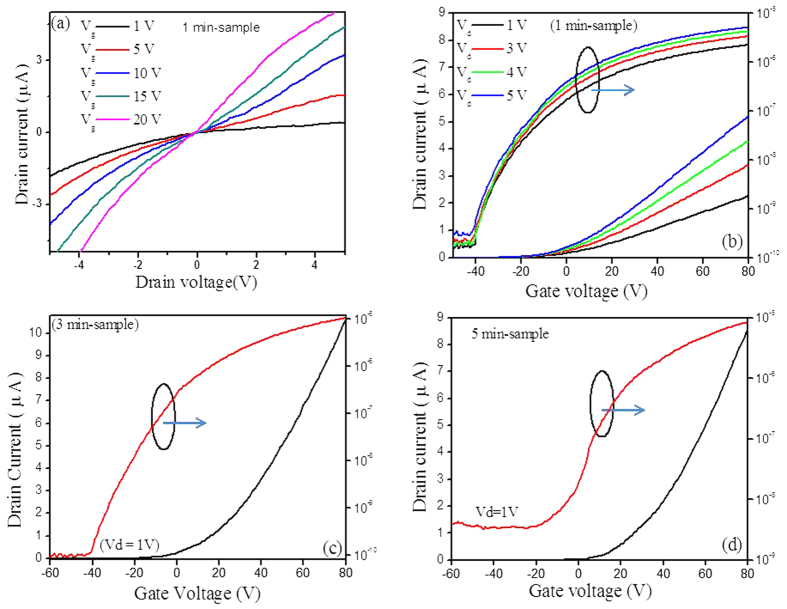
**(a)** I_d_–V_d_ of MoS_2_ FET of 1-min sample; **(b)** I_d_–V_g_ of MoS_2_ FET (1 min-sample) at V_d_ = 1, 3, 4 and 5 V; **(c**,**d)** I_d_–V_g_ of MoS_2_ FETs, 3 min-sample (**c**), and 5 min-sample (**d**) at fixed V_d _= 1V.

We have fabricated MoS_2_ FETs and performed I–V measurement to investigate electrical properties. The schematic diagram of MoS_2_ FET structure is given in Figure S15a. The active areas of FETs were defined during the sputtering process using a metal-shadow mask. As-sputtered MoS_2_ film exhibited very high resistance in the range between 16 GΩ and 0.2 GΩ. I_d_–V_g_ and I_d_–V_d_ plots of these devices are presented in Figure S14a–f. Our previous results showed that as-sputtered MoS_2_ at RT are amorphous structure and are oxidized. As a result, as-sputtered film can exhibit in high channel resistance and low current and mobility^54^,^55^. Figure 8a shows that I_d_–V_d_ curves of the 1 min-sample with respect to the back-gate voltages. Figure 8b shows the transfer characteristics of the annealed bilayer MoS_2_ FET (1 min-sample). The field-effect mobility was extracted based on the slope of ΔI_d_/ΔV_g_ fitted to the linear regime of the transfer curves using the following equation:




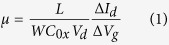




where W is the width of the channel (200 μm) L is the length of the channel (2300 μm), C_ox_ is the capacitance per unit area of the gate dielectric (1.15 × 10^−8^ F/cm^2^), V_d_ is the applied drain voltage (V_d_ = 1 V), and ΔI_d_/ΔV_g_ is the slope of the linear part of the transfer plot (I_d_–V_g_), or transconductance. The extracted transconductance, field-effect mobility, and on/off current ratio is ~2.9 × 10^−8^ S, 29 cm^2^/Vs and ~10^4^, respectively, at V_d_ = 1V. The linear drain current and the transconductance values at V_d_ = 1V are displayed in the Figure S15b. The transfer characteristics and I_d_–V_d_ curves for few-layer MoS_2_ FETs (3 and 5 min-sample) are shown in Figure S15c,d. The extracted transconductance values are ~1.81 × 10^−7^ S and ~1.73 × 10^−7^ S for the 3 min and 5 min-sample, respectively, which are ~6 times greater than bilayer MoS_2_ (1 min-sample). The current on/off ratio values are ~2 × 10^3^ −4 × 10^4^ for few-layer MoS_2_ FETs. The extracted field-effect mobility is ~181and ~173 cm^2^/Vs for 3 min-sample and 5 min-sample, respectively.

Table 1 compares field-effect mobility and I_on_/I_off_ values of our results with previously reported MoS_2_ FETs. A significant enhancement can be noted in our MoS_2_ FETs. It is interesting to compare with the recent reported mobility of ~12 cm^2^/Vs for thin MoS_2_ film, but the mobility decreased significantly to ~0.44 cm^2^/Vs for ~6.4 nm-MoS_2_ due to the incomplete transition of MoS_2_ from Mo^29^. To the best of our knowledge, our bilayer MoS_2_ FETs have higher mobility than any of latest results: exfoliated monolayer MoS_2_ FETs of 0.1–10 cm^2^/Vs, 10–15 cm^2^/Vs for exfoliated bilayer MoS_2_^2^, and ~17 cm^2^/Vs for CVD-grown single crystal bilayer MoS_2_^56^. It should be noted that some reports exhibiting very high mobility values for MoS_2_ film in Table 1 is due to the substrate effect such as sapphire or high-k gate oxide effect.

Besides, the mobility (173–181 cm^2^/Vs) of our few-layer MoS_2_ is the highest value ever for any MoS_2_ FETs with SiO_2_ gate dielectrics. Ayari *et al*.^31^ reported 10~50 cm^2^/Vs of mobility from single crystal exfoliated MoS_2_ flakes with 8~40 nm thickness. Our sputtered-MoS_2_ films have small grain sizes, which are smaller compared with an exfoliated MoS_2_. An important question then remains, what could be the possible mechanism for the high mobility behavior of our MoS_2_ film? For current 2D crystal materials, electron mobility is mostly dominated by charged impurity scattering, and the mobility values achieved to date are far below the intrinsic potential in these materials^52^.

We think that the high mobility behavior of our film could be attributed to low charged impurities of our film and dielectric screening effect by the interfacial MoO_x_Si_y_ layer. In our process, MoS_2_ films were directly sputtered on SiO_2_/Si substrate at high vacuum and transistors were fabricated without transfer step, while the conventional CVD-grown MoS_2_, except exfoliated MoS_2_, usually needs the wet-transfer process onto a desired dielectric substrate and it make high contamination. Since sputtering process is performed in a high vacuum chamber, the chemical residues and gaseous adsorbates could be minimized. In addition, the dielectric surface dangling bonds could be also minimized due to a strong interaction of Mo and O on SiO_2_ of the interfacial layer. Thus, low charged impurities could reduce the Coulomb scattering, resulting in high mobility values in the sputtered-MoS_2_^53^.

It is also well known that a bulk α-MoO_3_ possesses very high relative dielectric constants (>500 for α-MoO_3_)^57^. And the dielectric constants of an atomically thin α-MoO_3_ is still high even though it is low compared with its bulk value^58^. Thus, the MoO_x_Si_y_ could reduce Coulomb scattering effects due to its high-k value as well as low dielectric dangling bonds. We have also prepared MoO_3_ film on SiO_2_/Si substrate via a reactive sputtering using Mo target. XPS data of the sulfurized MoS_2_ from Mo target also have the MoO_3_ peak similar to the previous results (Figure S16). The as-sputtered MoO_3_ exhibited very high resistance due to a wide bandgap of the material. On the other hand, the sulfurized few-layer MoS_2_ FETs (from MoO_3_) exhibited high mobility values (~44 cm^2^/Vs) (Figure S17). This experiment also supports our hypothesis. The fact that few-layer MoS_2_ has much higher mobility value than that of bilayer MoS_2_ reflects a critical role of Coulomb interaction distance upon the mobility values since thicker film has longer interaction distance. We compared hysteresis in transfer curves of FETs made by exfoliated-MoS_2_, CVD-grown MoS_2_, and sputtered-MoS_2_ (Figures S18 and S19). It is well known that the origin of hysteresis of conventional FETs is due to the trapping and detrapping of carriers^59^. The trapping and detrapping can occur at the interface of the MoS_2_/SiO_2_ or at the top surface of MoS_2_. Imperfect interface between MoS_2_ and SiO_2_ such as foreign molecules trapped at the interface or dielectric dangling bonds could contribute to the interface trap of MoS_2_/SiO_2_. Chemical residues or moisture or oxygen on the MoS_2_ surface could contribute the charge trapping at the top surface of MoS_2_ film. Water and oxygen in ambient environment also have been reported to cause hysteresis of MoS_2_ FETs due to the charge transferring on MoS_2_ top surface^59^.

We compared the hysteresis under vacuum environment to prevent such extrinsic and environmental effects and focus on the trapping at the MoS_2_/SiO_2_ interface^60^. The exfoliated-MoS_2_ and CVD-grown MoS_2_ exhibited large hysteresis in there I_d_–V_g_ curves. On the contrary, the sputtered-MoS_2_ film exhibited small hysteresis. Such improvement in the hysteresis can be attributed to the small trap at the MoS_2_/SiO_2_ interface of the sputtered-MoS_2_ film. It is thought that charge scattering due to charge trapping is reduced due to the interfacial layer and enhance the mobility behavior of our sputtered-MoS_2_ film.

## Conclusions

We have successfully demonstrated the growth of large-area and continuous bilayer to few-layer MoS_2_ on SiO_2_/Si substrate via RF sputtering combined with the post-deposition annealing process. The crystalline quality of the as-sputtered films was substantially improved via annealing at 700 °C in the sulfur and argon environment. The bilayer MoS_2_ FETs exhibited a high field-effect mobility of ~29 cm^2^/Vs and an on/off ratio of ~10^4^. The mobility value of our bilayer MoS_2_ FETs is larger than any of latest results of single to bilayer MoS_2_ grown on a SiO_2_/Si substrate with a SiO_2_ gate oxide. The mobility for few-layer MoS_2_ FETs increased to ~173–181 cm^2^/Vs. Our few-layer MoS_2_ FETs exhibited the highest mobility value ever for any MoS_2_ FETs with a SiO_2_ gate oxide. It is presumed that the high mobility behavior of our film could be attributed to low charged impurities of our film and dielectric screening effect by the interfacial MoO_x_Si_y_ layer. The combined synthesis route of MoS_2_-RF sputtering with the post-deposition annealing process could open up the possibility of mass and batch production of MoS_2_ film. We believe our proposed strategy will pave the way for applications of MoS_2_ in future electronics and optoelectronics.

## Method

The various sizes of SiO_2_ (300 nm)/Si substrates ranging from 1 × 1 cm^2^ to 3 × 3 cm^2^ were used for the film preparation process. All the substrates were cleaned in acetone, methanol, isopropyl alcohol (IPA) solution and deionized (DI) water and then dried and baked for 5 min. After loading the SiO_2_/Si substrates into a sputtering chamber, the chamber was vacuumed at 1 × 10^−6^ Torr. Before the deposition process, the MoS_2_ target (99.99% purity) was pre-sputtered in a pure argon (Ar) atmosphere for 5 min in order to remove the oxide layer on the surface of the target. The MoS_2_ films were sputtered at various temperatures: RT, 200, 300, 400 and 500 °C. The chamber pressure was maintained at 10 mTorr during the deposition in an Ar atmosphere, and the RF power was kept constant at 25 W for 1 min. The temperature variation in the chamber was monitored through a thermocouple. The as-sputtered MoS_2_ films were post-annealed at 700 °C under Ar and sulfur environment to improve the crystalline quality of the films. The as-deposited films were placed in an annealing chamber and heated up to 700 °C for 30 min, 1 hour, 2 hours, and 3 hours. The carrier gas flow rate was maintained at 100 sccm, and the pressure of chamber was kept at 2 × 10^−2^ Torr.

### Fabrication of the MoS_2_ FET devices

The active area of MoS_2_ FET was formed during sputtering using a shadow mask. This kind of shadow mask is to avoid any chemical contamination by traditional active area preparation route of photolithography or electron-beam lithography. The metal contacts of 6 nm-Ti/30 nm-Au were prepared by evaporation. After making the electrode contacts, the devices were annealed at 200 °C for 2 hour in a vacuum tube furnace with 100 sccm Ar flow. After the annealing, the resistance of devices decreased significantly. The electrical properties of the fabricated MoS_2_ transistors were measured using the 2 probe method at room temperature in a vacuum chamber to avoid oxidation.

### Characterization details of MoS_2_ films

Synthesized MoS_2_ films were analyzed by Raman spectroscopy (Renishaw invia RE04, 512 nm Ar laser) with a spot size of 1 μm and a scan speed of 30 seconds. A Si substrate with a Raman peak of 520 cm^−1^ was used for calibration. X-ray photoelectron spectroscopy (XPS) (PHI 5000 Versa Probe, 25W Al Kα, 6.7 × 10^−8^ Pa) and photoluminescense (PL) with a 512 nm wavelength was used. Laser radiation of PL was focused onto the MoS_2_ film with a spot-size of around 1 μm. FE-SEM (HITACHI S-4700) and atomic force microscopy (AFM) (Vecco Dimension 3100) were used to check the morphology and thickness of the films. TEM samples were prepared using lacey-carbon Cu grid. The atomic structure of MoS_2_ thin films was characterized by a JEOL-2010F TEM with an accelerating voltage of 200 keV. Image acquisition and processing (FFT, IFFT, etc.) were performed using the Gatan Digital Micrograph software (Gatan Microscopy Suite 2.0). The crystallinity of the film was characterized by in-plan X-ray diffraction (XRD, Rigaku) with Cu-Kα radiation operated at 50 KV and 300 mA.

## Additional Information

**How to cite this article**: Hussain, S. *et al*. Large-area, continuous and high electrical performances of bilayer to few layers MoS_2_ fabricated by RF sputtering via post-deposition annealing method. *Sci. Rep.* **6**, 30791; doi: 10.1038/srep30791 (2016).

## Supplementary Material

Supplementary Information

## Figures and Tables

**Table 1:** Literature values of room temperature field-effect mobility for MoS_2_ FETs grown by various methods.

S. No.	Growth method	I_on_/I_off_	Mobility [cm^2^/Vs]	Ref.
1	Sputtering (MoS_2_) + CVD	~10^4^	~29 (~1.4 nm) ~173–181 (~3.8~6 nm)	This work
2	CVD (MoO_3_ + S)	~10^7^	24	Appl.Phys. Lett., 106 (2015) 062101
3	Sputtering (MoS_2_)	~10^3^	12.2	Nanoscale, 2015,7, 2497–2503
4	MoO_3_ powder + Mo substrate+ CVD		192	Appl. Phys. Lett., 105 (2014) 072105
5	Ebeam (Mo) + CVD		12±2	Appl. Phys. Lett., 102 (2013) 252108.
6	Sputtering (Mo)+ CVD	~1.5 × 10^6^ ~5 × 10^4^	12 (~1.1 nm) 0.44 (~6.4 nm)	ACS Appl. Mater. Interfaces, 2014, 6 (23), 21215–21222
7	CVD (MoCl_5_ + S)	~10^4^~10^5^	0.003–0.03	Scientific Reports 3 : 1866 DOI: 10.1038/srep01866
8	CVD (MoO_3_ + S) on rGO	~10^4^	0.02	Adv. Mater. 2012, 24, 2320–2325
9	CVD (MoO_3_ + S)	~10^6^	2~7	ACS Nano, 2014, 8 (6), 6024–6030
10	Ebeam (Mo) + CVD		0.004~0.04	Small 2012, 8, 966.
11	CVD (MoO_3_ + S)	~10^8^	17	Appl. Phys. Lett. 100, 123104 (2012)
12	CVD (MoO_3_ + S)	~10^4^ ~10^6^	0.1~0.7	J. Amer. Chem. Society 2013, 135, 5304.
13	CVD (MoO_3_ + S)	~10^3^	0.09	Nano Research 2014, 7 (12) : 1759–1768
14	Thermal (MoO3)+ CVD on sapphire	~10^5^	~0.8	Nanoscale, 2012,4, 6637–6641
15	Thermolysis of (NH_4_)_2_MoS_4_	~10^5^	4.7~6	Nano Lett., 2012, 12 (3), 1538–1544
16	Exfoliated (electrochemical)	~10^6^	1.2	ACS Nano, 2014, 8 (7), 6902–6910
17	CVD (H_2_S +Mo)	~10^5^	0.12	Nanoscale, 2014,6, 2821–2826
18	Mo(CO)_6_ + (C_2_H_5_)2S	~10^4^	30	Nature, 2015, 520, 656–660
19	CVD (MoO_3_ + S)	~10^6^	3.6 (1L), 8.2 (2L), 15.6 (3L)	Nanoscale, 2015,7, 1688–1695
20	CVD(MoO_3_ + S)	10^5^~10^7^	~3 to 4	Nat. Mater., 2013, 12, 554–561.
