# Supplementary material for: Large-area, continuous and high electrical performances of bilayer to few layers MoS2 fabricated by RF sputtering via post-deposition annealing method
Source: Sci Rep. 2016 Aug 5;6:30791. doi: 10.1038/srep30791 (PMC4974610; doi:10.1038/srep30791)
Supplement: Supplementary Information [file srep30791-s1.doc]

Electronic Supporting Information

**Large-area, continuous and high electrical performances of bilayer to few layers MoS_2_ fabricated by RF sputtering via post-deposition annealing method**

Sajjad Hussain^a,b^, Jai Singh^c^, Dhanasekaran Vikraman^d^, Arun Kumar Singh^e^, Muhammad Zahir Iqbal^e^, Muhammad Farooq Khan^e^, Pushpendra Kumar^f^, Dong-Chul Choi^a,b^, Wooseok Song^h^, Ki-Seok An^h^ , Jonghwa Eom^e^, Wan-Gyu Lee*^g^, and Jongwan Jung*^a,b^

^a^Graphene Research Institute, Sejong University, Seoul 143-747, Korea

^b^Faculty of Nanotechnology & Advanced Materials Engineering and Graphene Research Institute, Sejong University, Seoul 143-747, Korea.

^c^Dr. H. S. Gour Central University, Sagar, M.P.-470003, India

^d^Division of Energy Systems Research, Ajou University, Suwon 443-749, Republic of Korea.

^e^Department of Physics and Graphene Research Institute, Sejong University, Seoul 143-747, Korea.

^f^Institute of Atomic and Molecular Sciences, Academia Sinica, Taipei, 10617, Taiwan.

^g^National Nano Fab Center, Daejeon, Korea.

^h^Thin Film Materials Research Group, Korea Research Institute of Chemical Technology, Daejon 305-600, Korea

***Corresponding authors E-mail:** (J.J) [jwjung@sejong.ac.kr](mailto:jwjung@sejong.ac.kr), (W. L) [wangyulee@nnfc.re.kr](mailto:wangyulee@nnfc.re.kr)

**Figure S1.** (a) Raman spectra of as-sputtered MoS_2_ films prepared at 1, 3 and 5 min sputtering time. (b) Raman spectra of 1, 3 and 5 min samples annealed at 700°C (c) Raman spectra of as-sputtered MoS_2_ films at RT and 400^o^C, and annealed MoS_2_ film (sputtered at RT and annealed at 700° C under Ar and sulfur environment for 1 hour); (d) Expanded view of MoO_3_ related scattering lines.

**Figure S2.** Raman mapping analysis of 3 min and 5 min-sample. All the Raman mapping is performed over an area of 30 µm × 30 µm. **(a)** E^1^_2g_ mode position appeared at ~382.23-382.33 cm^-1^ (with a standard deviation 0.05 cm^-1^) and **(b)** A_1g_ mode appeared at ~407.29-407.39 cm^-1^(with a standard deviation 0.045 cm^-1^) for 3-min sample. **(c)** The measured frequencies difference ∆k are in a range of ~24.96-25.16 cm^-1^ (with a standard deviation 0.066 cm^-1^); **(d)** E^1^_2g_ mode appeared at ~380.63 – 380.73 cm^-1^ (with a standard deviation 0.05 cm^-1^) and **(e)** A_1g_ mode appeared at ~408.29- 408.39 cm^-1^ (with a standard deviation 0.047 cm^-1^) for 5-min sample. **(f)** The measured frequencies difference ∆k are in a range of 27.56 – 27.76 cm^-1^ (with a standard deviation 0.070 cm^-1^).

**Table S1** Statistically analysis of Raman mapping of 1, 3 and 5 min. growth of MoS_2_ film.

|  | | **1 min. growth** | **3 min. growth** | **5 min. growth** |
| --- | --- | --- | --- | --- |
| **E^1^_2g_ mode** | Average | 384.857 | 382.283 | 380.68 |
|  | Max | 384.92 | 382.23 | 380.63 |
|  | Min | 384.82 | 382.33 | 380.73 |
|  | Standard deviation | 0.048 | 0.05 | 0.050 |
| **A_1g_ mode** | Average | 405.248 | 407.318 | 408.32 |
|  | Max | 405.29 | 407.39 | 408.39 |
|  | Min | 405.19 | 407.29 | 408.29 |
|  | Standard deviation | 0.049 | 0.045 | 0.0470 |
| **∆ k** | Average | 20.390 | 25.035 | 27.642 |
|  | Max | 20.47 | 25.16 | 27.76 |
|  | Min | 20.27 | 24.96 | 27.56 |
|  | Standard deviation | 0.066 | 0.066 | 0.070 |

**Figure S3.** Raman spectra of as-synthesized MoS_2_ at various substrate temperatures from 200° to 500°C. Peak difference (Δk) values are ~31, ∼30, ~28, and ∼26 cm^-1^ for substrate temperature of 200, 300, 400, and 500°C, respectively.

**Figure S4.** In plane XRD patterns of as-sputtered and 700°C-annealed MoS_2_ films prepared at different sputter time: (a) 1 min, (b) 3 min, and (c) 5 min.

**Figure S5.** Typical X-ray diffraction patterns of as-synthesized MoS_2_ at various substrate temperatures from 200° to 500°C. The highly intense sharp peak is exhibited at 33.44^o^ diffraction angle corresponding to the (100) lattice orientation, which is related to the substrate. A weak peak is observed at a 14.10^o^ diffraction angle corresponding to the (002) lattice orientation.

**Figure S6.** XPS spectra of as-sputtered MoS_2_ films (sputtered at 1, 3 and 5 min).

**Figure S7.** XPS survey scan for (a) as-sputtered and (b) annealed MoS_2_ films. (c) Depth profile of XPS survey scan for 5min-sample.

**Figure S8.** XPS Depth profile of few-layer MoS_2_ (30 min-sample). **(a)** Mo 3d core peaks as a function of etching time. Mo core level peak position are observed at 232.4 and 229.3 eV corresponding to Mo^4+^ 3d_3/2_ and 3d_5/2_ atoms, respectively, for MoS_2_ film at 0 etching time. This position is shifted to the lower energy. **(b)** Sulfur core peaks as a function of etching time. Sulfur peaks disappear after 2160 sec but Mo peaks still exist. **(c & d)** O 1s and S 2p peak depth profile with the etching time.


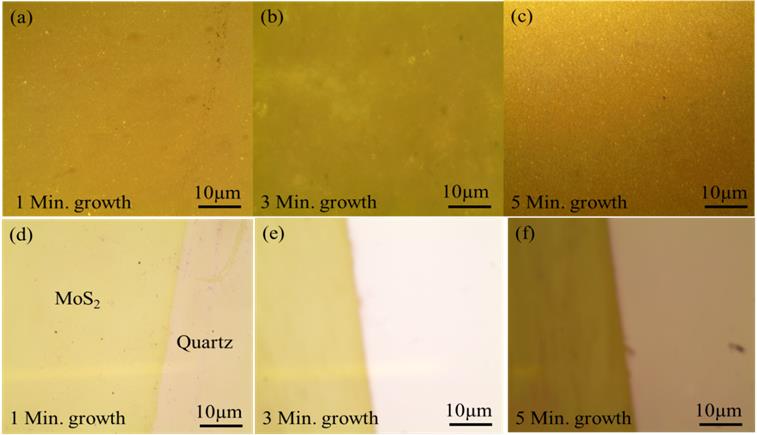


**Figure S9.** (a-f) Optical images of MoS_2_ films (annealed at 700^o^C) grown onto sapphire and quartz substrate.

**Figure 10. (a)** PL spectra of as-sputtered MoS_2_ films prepared at different sputter time (1, 3 and 5 min) **(b)** PL spectra of annealed-MoS_2_ films prepared at different sputter time (annealing time was fixed to 3 hour) **(c)** PL spectra of annealed-MoS_2_ films annealed at different annealing time (sputter time was fixed to 1 min)

**
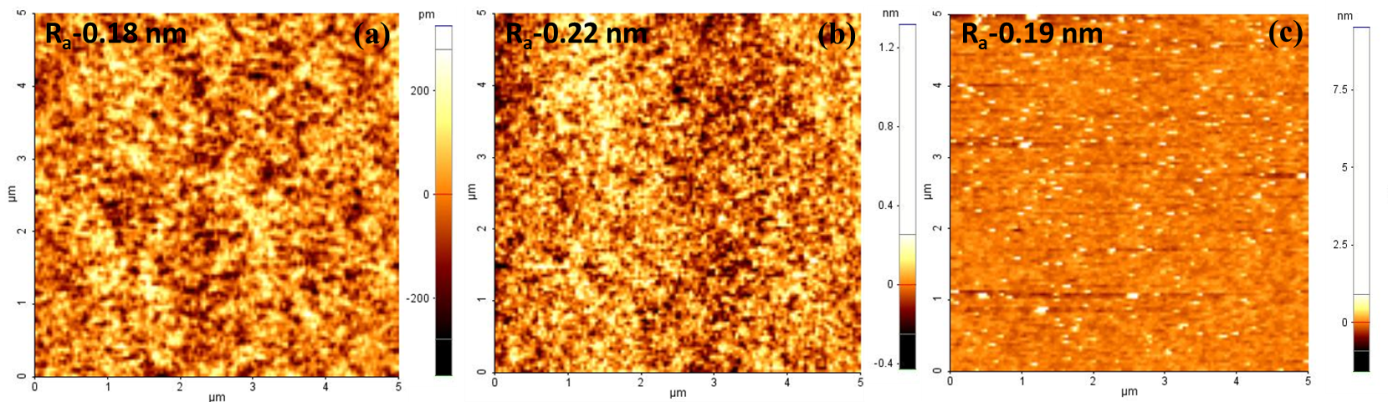
**

**Figure S11.** (a-c) AFM topographical images of as-sputtered MoS_2_ films sputtered at different time (a) 1 min (b) 3 min and (c) 5 min.

**Figure S12.** Large area HRTEM image for 1 min-sample. HRTEM images of (b) 3 min-sample (c) 5 min-sample

**Figure S13.** (a) Large-area MoS_2_ films on SiO_2_ substrates (approximately 9 cmx1cm), (b-d) Raman peak positions of E^1^_2g_, A_1g_ bands and the peak differences (Δk) plotted at ten different points for the 1-min, 3-min, and 5-min sputtered MoS_2_ films.

**Figure S14.** Electrical properties of MoS_2_ FETs using as-sputtered MoS_2_ films. (a) (b) As-sputtered for 1 min, (c)(d) As-sputtered for 3 min **, (e) (f)** As-sputtered for 5 min **(a)(c)(e)**  I_d_ - V_g_ at V_d_ = 1V (b) (d) (f) I_d_ -V_d_**;**


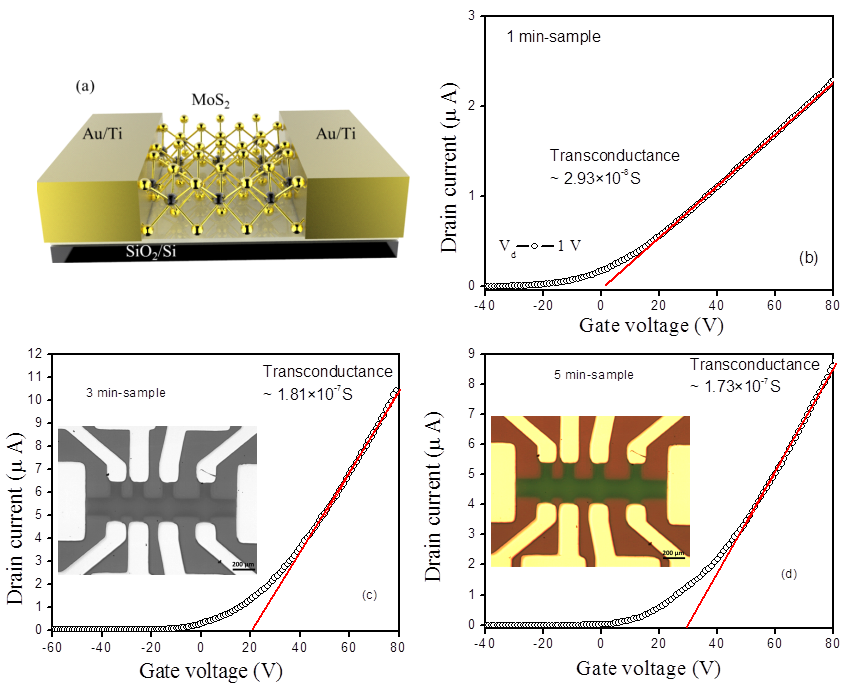


**Figure S15.** (a) Schematic diagram of FETs (b-d) Linear I_d_-V_g_ of MoS_2_ FETs (V_d_ = 1V). Red line is a linear-fit (transconductance) to the I_d_. Mobility value is calculated from the transconductance value. The extracted transconductance values of 1, 3 and 5 min samples are 2.93×10^-8^ S, 1.81×10^-7^ S and 1.73×10^-7^ S, respectively.(Inside,: optical image of as-fabrication FET device with different filters for 3 and 5 min samples)

**Figure S16.** XPS spectra of MoS_2_ (annealed from the as-sputtered MoO_3_) and the as-sputtered MoO_3_.

**Figure S17.** Linear I_d_-V_g_ of MoS_2_ FET (sulfurized from the as-sputtered MoO_3_ film). A Mo target was reactive-sputtered at 120 sec under an oxygen gas, and then, as-sputtered MoO_3_ was subjected to anneal in sulfur and Ar atmosphere. Red line is a linear-fit (transconductance) to the I_d_. The transconductance value is ~1.4×10^-7^ S, and estimated mobility value is ~ 44 cm^2^/Vs.

**Figure S18.** Comparison of hysteresis in transfer curves of few-layer MoS_2_ FETs prepared by (a) exfoliation method (c) CVD method (e) sputtering method. (b) AFM step height profile of exfoliated sample (~ 6 nm). (d) Raman spectra of CVD-MoS_2_ (peak spacing is corresponds to few-layer MoS_2_.) All the film thickness is approximately 6 nm. Drain bias was set to 1V, and the scan rate was fixed to 1V/sec (-60V to 80V).

**Figure S19.** Hysteresis in transfer curves of sputtered- MoS_2_ FETs. Drain bias was fixed to 1V, and sweeping rate is 1V/sec from -60 to 17 V.
